# Attention U-Net-Based Segmentation and Hybrid Classification for Detection of Circulating Tumor-Associated Cells

**DOI:** 10.3390/cancers18162691

**Published:** 2026-08-20

**Authors:** Massimo Cristofanilli, Sewanti Limaye, Nitesh Rohatgi, Timothy Crook, Humaid O. Al-Shamsi, Andrew Gaya, Raymond Page, Aditya Shreenivas, Darshana Patil, Vineet Datta, Dadasaheb Akolkar, Stefan Schuster, Prashant Kumar, Shoeb Patel, Pradyumna Shejwalkar, Snehal Golar, Ajay Srinivasan, Rajan Datar

**Affiliations:** 1 Department of Medical Oncology, Weill Cornell Medicine and New York Presbyterian Hospital, New York, NY 10065, USA; mac9795@med.cornell.edu; 2 Department of Medical and Precision Oncology, Sir HN Reliance Foundation Hospital and Research Centre, Mumbai 400004, Maharashtra, India; sewanti.limaye@rfhospital.org; 3 Department of Medical Oncology, Fortis Memorial Research Institute, Gurugram 122003, Haryana, India; docrohatgi@gmail.com; 4 Department of Medical Oncology, Cromwell Hospital, London SW5 0TU, UK; tr.crook@gmail.com; 5 Burjeel Cancer Institute, Burjeel Medical City, Abu Dhabi 114448, United Arab Emirates; humaid.al-shamsi@medportal.ca; 6 Department of Medical Oncology, Dana-Farber Cancer Institute, Harvard Medical School, Boston, MA 02215, USA; 7 College of Medicine, Ras Al Khaimah Medical and Health Sciences University, Ras Al Khaimah 11172, United Arab Emirates; 8 Department of Clinical Oncology, Cromwell Hospital, London SW5 0TU, UK; andygaya@icloud.com; 9 Department of Biomedical Engineering, Worcester Polytechnic Institute, Worcester, MA 01609, USA; rpage@wpi.edu; 10 Department of Medical Oncology, City of Hope National Medical Center, Duarte, CA 91010, USA; ashreenivas@coh.org; 11 Datar Cancer Genetics, Nasik 422010, Maharashtra, India; drvineetdatta@datarpgx.com (V.D.); dadasaheb.akolkar@datarpgx.com (D.A.); prashant.agrawal@datarpgx.com (P.K.); shoeb.patel@datarpgx.com (S.P.); pradyumna.shejwalkar@datarpgx.in (P.S.); snehal.golar@datarpgx.in (S.G.); ajays@datarpgx.org (A.S.); rajandatar@datarpgx.com (R.D.); 12 Datar Cancer Genetics Europe GmbH, 95447 Bayreuth, Germany; drstefanschuster@datarpgx.com

**Keywords:** circulating tumor cells (CTCs), circulating tumor associated cells (CTACs), multi-cancer early detection (MCED), liquid biopsy, U-Net architecture, attention U-Net, image based cell detection, pixel-level segmentation, artificial intelligence, skip connections

## Abstract

**Background/Objectives:** Circulating tumor-associated cells (CTACs) are rare among peripheral blood nucleated cells (PBNCs), creating a challenge for image-based multi-cancer detection. We evaluated a predefined CTAC-detection pipeline incorporating Attention U-Net segmentation, post-processing, cytological feature extraction, and Random Forest classification. **Methods:** Model suitability was explored in asymptomatic individuals and patients with advanced solid tumors. Clinical performance was assessed in a case–control cohort of therapy-naive stage I/II cancers, benign conditions, and asymptomatic individuals, followed by four prospective cohort evaluations performed within the same laboratory and imaging workflow: recurrent cancer with low radiological tumor burden, peri-operative solid tumors, suspected cancer, and asymptomatic screening. PBNCs were stained with EpCAM/Hoechst 33342 and imaged. Pathologists’ review established ground truth annotations. **Results:** The model had 90.68% sensitivity and 99.53% specificity in the exploratory study. In the case–control cohort, sensitivity was 88.65% in therapy-naive stage I/II cancers, while specificity was 78.95% in benign conditions and >99.9% in asymptomatic individuals. In the prospective cohorts, CTAC detection sensitivity was 91.96% in pretreated low tumor burden cases; CTACs were detected in 100% of pre-surgery specimens and 29.41% of post-surgery specimens; and in suspected cancer cases, the Positive Predictive Value (PPV) and Negative Predictive Value (NPV) were 96.34% and 32.35%, respectively. In the asymptomatic screening cohort, 44/7183 participants were CTAC-positive; 16 had confirmed Stage I/II cancer, 10 had no radiologically detectable disease at the available assessment, and 18 remained unresolved. The conservative lower-bound PPV was 36.36%, and the NPV was 99.97%; estimates remain provisional pending complete follow-up. **Conclusions:** The integrated Attention U-Net/feature-based classification pipeline demonstrated consistent CTAC detection across the evaluated cohorts and supports its potential clinical utility for cancer detection.

## Simple Summary

Blood-based cancer detection is challenged by the rarity of tumor-associated cells among abundant peripheral blood nucleated cells. This study evaluates a predefined image-analysis pipeline that combines dual-channel fluorescence microscopy, an Attention U-Net segmentation stage, CTAC-specific post-processing, extraction of 64 predefined cytological features, and a Random Forest classifier. The attention gates follow established Attention U-Net principles; the study-specific contribution is the integration of these components for CTAC detection and their evaluation across case–control and prospective cohorts. The results support the potential clinical utility of the integrated framework for CTAC detection.

## 1. Introduction

Circulating tumor cells (CTCs) are cancer cells that detach from malignant tumors, enter the bloodstream, and potentially lead to distant metastasis [1,2]. CTCs have been recognized as potential biomarkers for early cancer detection [3], disease monitoring [4], and treatment response assessment [5]. CTCs are clinically relevant analytes and their detection and characterization may aid cancer diagnostics, prognostication, and treatment monitoring [6,7]. We have previously described circulating tumor-associated cells (CTACs) that include classical CTCs and other tumor-associated cells that are positive for epithelial markers, irrespective of CD45 status [8]. Assessing CTACs rather than only CTCs improves sensitivity by capturing epithelial marker-positive cells that may be missed by CD45-restricted definitions, thus better reflecting tumor heterogeneity and tumor-immune interactions that drive tumorigenic mechanisms. Consequently, CTACs provide a more comprehensive representation of tumor biology. We have also described the process for identification of CTACs via immunostaining followed by image analysis [9]. Conventional CTAC detection via immunostaining along with microscopy or flow cytometry faces sensitivity limitations in addition to labor-intensive protocols and varied specificity due to subjective manual interpretation [10,11].

Artificial intelligence (AI)-based automation of cell detection and classification can qualitatively and quantitatively improve medical image analysis [12,13]. However, conventional convolutional neural networks (CNNs) often struggle with the fine-grained discrimination required for identifying rare CTACs among the exponentially higher background of normal (i.e., non-malignant) peripheral blood nucleated cells (PBNCs) [14]. This challenge is compounded by subtle differences in morphological and marker expression characteristics among CTACs [15].

The U-Net architecture, originally developed for biomedical image segmentation [16], offers a solution to the skewed signal-to-noise ratio in imaging. The encoder–decoder structure with skip connections preserves spatial information while enabling deep feature extraction, making it particularly suitable for tasks requiring pixel-level precision [17]. Despite these advantages, the application of U-Net architectures to CTAC detection from fluorescence microscopy images remains underexplored. Attention gating has been incorporated into U-Net architectures to suppress irrelevant activations and improve localization of task-relevant regions in biomedical segmentation [18,19,20,21]. Related attention-based U-Net variants have also been evaluated for cell and nuclei segmentation in histopathology. The present work adopts the established Attention U-Net gating principles.

We developed a predefined integrated pipeline for automated CTAC detection from PBNCs stained with EpCAM and the cell-permeable nuclear dye Hoechst 33342. The segmentation module follows the established Attention U-Net encoder–decoder principle with attention-gated skip connections. Study-specific processing comprises dual-channel input, CTAC-oriented post-processing, extraction of 64 cytological features, and downstream Random Forest classification. In this manuscript, we evaluate the performance of the complete integrated framework across controlled and prospective clinical cohorts. The study objective did not include a comparative assessment of various U-Net models but was intended for a standalone assessment of the complete (integrated/end-to-end) design. We do not seek to attribute the observed performance to any individual model-development component.

## 2. Materials and Methods

### 2.1. Study Design and Subjects

All studies and sub-studies were conducted after approval by the Institutional Ethics Committee (IEC) of the Study Sponsor (Datar Cancer Genetics, DCG) and in accordance with applicable regulatory requirements and the Declaration of Helsinki. Study procedures included the collection of biological specimens (peripheral blood) from (a) asymptomatic individuals with no prior history of cancer diagnosis, (b) individuals with histopathologically confirmed non-malignant (benign) conditions, (c) individuals with histopathologically confirmed cancers, and (d) individuals with symptoms or clinical laboratory or radiological findings suspected of cancer. No individual received any treatment or underwent any invasive procedures solely for the purpose of this study—any treatments or invasive procedures referenced in this study were a part of the respective standard of care (SoC) management pathways. Informed consent was obtained from all study participants for the collection and use of deidentified specimens for research purposes and the publication of deidentified data and findings. In addition, the study also evaluated leftover blood specimens from individuals who had previously utilized the study sponsor’s oncology diagnostic services and had consented to the use of leftover deidentified specimens for research and scientific purposes and the publication of deidentified data and findings. The study sponsor’s test order forms include an IEC-approved (optional) consent statement for the research use of deidentified leftover specimens and publication of deidentified data.

### 2.2. Benign and Confounding Condition Representation

To minimize spectrum bias and evaluate potential sources of false positive results, we incorporated a cohort of individuals with benign clinical conditions and non-malignant diagnoses. These included subjects with benign tumors and non-neoplastic conditions that are commonly encountered during real-world cancer diagnostic workups. Such conditions can produce circulating cellular or nucleic acid signals that may mimic malignancy and therefore represent critical confounders in the evaluation of multi-cancer early detection (MCED) technologies. The benign cohort comprised individuals diagnosed with conditions including benign tumors, inflammatory diseases, autoimmune disorders, and other non-malignant pathological states. Where available, clinical diagnoses were confirmed through standard-of-care investigations, including imaging, laboratory testing, and histopathological evaluation. To further reflect real-world diagnostic pathways, the suspected-cancer cohort included individuals undergoing clinical evaluation for possible malignancy prior to histopathological confirmation. Blood samples were collected prior to definitive diagnostic biopsy or surgical intervention wherever feasible. This design enabled evaluation of assay performance in a pre-diagnostic clinical setting (“intent-to-test population”), which more closely reflects the intended clinical application of MCED technologies.

### 2.3. Mitigation of Spectrum and Verification Bias

Recognizing the methodological limitations frequently encountered in case–control validation studies of MCED assays, the present study incorporated both retrospective case–control cohorts and prospective cohorts reflecting real-world clinical use scenarios. Specifically, (a) a case–control cohort including cancer patients and individuals with benign conditions was used to establish analytical performance characteristics; (b) a suspected-cancer cohort representing an intent-to-test population undergoing diagnostic evaluation was included to assess performance in a pre-diagnostic clinical context; and (c) a large asymptomatic screening cohort was included to explore potential application in population-level cancer detection. Although not all screening participants underwent confirmatory diagnostic procedures, individuals with positive assay findings were advised to undergo further clinical evaluation and follow-up. This approach reflects pragmatic real-world implementation and acknowledges the potential for verification bias inherent in screening studies.

### 2.4. Sample Collection and Processing

**Figure 1:**
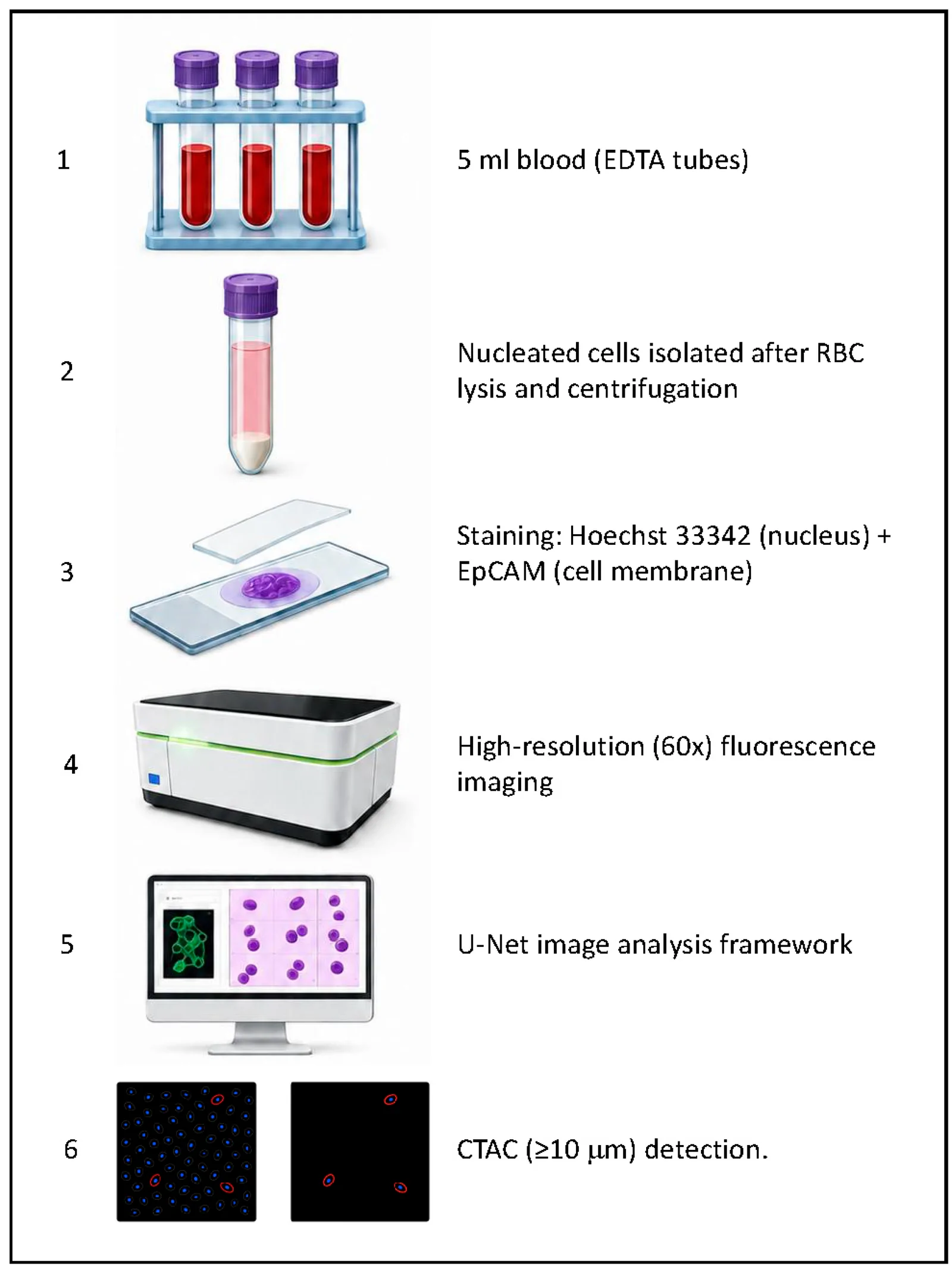
Sample processing workflow diagram showing the steps from blood collection to slide preparation for imaging.

Peripheral blood samples (~5 mL) were collected in EDTA vacutainers (Becton Dickinson and Company, Franklin Lakes, New Jersey, USA) from all participants by venous draw. All samples were processed within 72 h of collection. Initially, red blood cells (RBCs) were lysed using a commercial lysis buffer (BD Pharm Lyse™ BD Biosciences, San Jose, California, USA) according to the manufacturer’s instructions. Peripheral blood nucleated cells (PBNCs) were isolated by centrifugation at 400× *g* for 5 min at room temperature (RT). The cell pellet was washed twice with 1× phosphate-buffered saline (PBS) (HiMedia Laboratories Pvt. Ltd., Mumbai, India) and resuspended in 1 mL of 1× PBS. Cells were fixed with 4% paraformaldehyde (15 min, RT) (MP Biomedicals, LLC, Solon, Ohio, USA). Immunostaining of epithelial cell adhesion molecule (EPCAM) was performed using PE Vio 615-conjugated recombinant human anti-CD326 (Miltenyi Biotec, Bergisch Gladbach, Germany). Immunostained cells were mounted on adhesive slides (Superfrost Plus, Thermo Fisher, Waltham, Massachusetts, USA) and cover-slipped using anti-fade mounting medium (ProLong Gold, Invitrogen, Carlsbad, California, USA). High-resolution fluorescence microscopy images were acquired using an automated Zeiss Axio Imager 2 slide scanner (Carl Zeiss AG, Oberkochen, Germany) with an oil-immersion objective, using Hoechst 33342 and PE Vio 615 channels at specified wavelengths, and Z-stack acquisition. For each sample, multiple non-overlapping fields were captured, processed as maximum intensity projections, and stored as uncompressed TIFF files to preserve the dynamic range. All samples were processed in batches with positive and negative controls (Figure 1). All laboratory personnel were blinded to the clinical status of the samples during processing. Details of process reagents are provided in Supplementary Table S1.

### 2.5. Reference Controls

**Figure 2:**
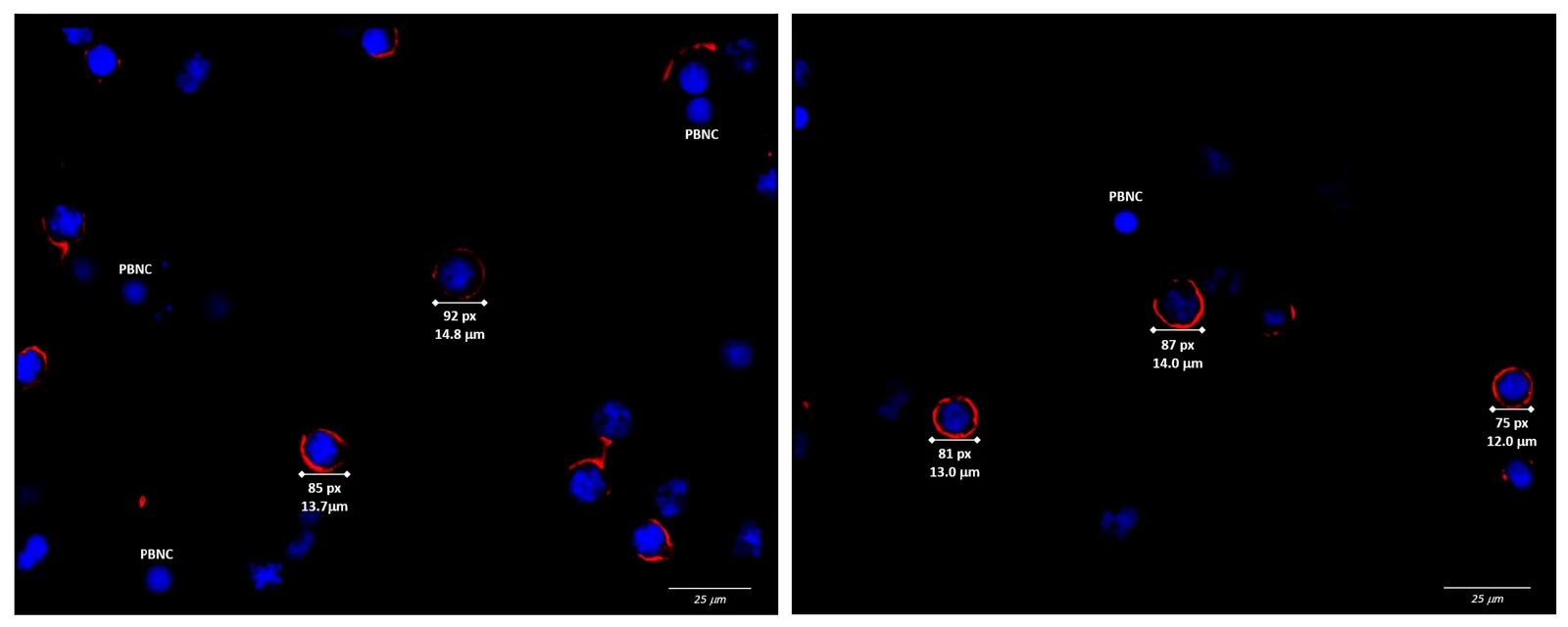
Representative images showing annotation of CTACs versus non-CTACs, highlighting the morphological and fluorescence characteristics used for discrimination. The scale bar (25 micron) is indicated at the bottom right. Explanatory schematics of processing logic are provided in Figure 3.

Contrived samples were generated using MCF-7 (breast carcinoma), SW-620 (colorectal adenocarcinoma), SKBR3 (breast carcinoma), A-549 (lung carcinoma), PL-45 (pancreatic cancer), OVCAR-3 (ovarian cancer), and PC-3 (prostate cancer) cell lines, all of which have well-characterized epithelial features and EPCAM expression (verified periodically by immunostaining). All cell lines underwent periodic mycoplasma testing and short tandem repeat (STR) profiling. Cells were cultured and harvested using standard techniques. Contrived specimens were generated by spiking 100–200 cells into 5 mL of healthy donor whole blood and immunostaining (and imaging) as described in the previous section. High-resolution images of biomarker-positive cells were used in initial model development and training and in analytical validation. Contrived specimens were processed in parallel with study samples according to the established protocols and served as positive controls. Representative images showing the annotation of CTACs versus non-CTACs, highlighting the morphological and fluorescence characteristics, are shown in Figure 2. The details of control cell lines and critical process reagents are provided in Supplementary Table S1.

### 2.6. Ground Truth Annotation

To establish ground truth for model training and evaluation, a subset of images was manually annotated by a panel of pathologists. The first step of annotation involved independent review of images by each pathologist using an in-house annotation software. In the next step, cellular boundaries were manually marked at the pixel level. Finally, the cells were classified as either CTACs or non-CTACs based on (a) EPCAM positivity (fluorescence signal localized to the cell membrane), (b) nuclear morphology (irregular nucleus on Hoechst 33342 staining), (c) cell size (diameter > 10 μm), and (d) nuclear-to-cytoplasmic ratio (N:C ≥ 0.7). Annotations with at least 90% agreement were considered in the final ground truth dataset. For discordant observations, the consensus was reached through joint review. A total of 2200 cells were annotated, including 1000 confirmed CTACs from cancer patients and 1200 non-CTACs from both cancer patients and asymptomatic individuals.

### 2.7. U-Net Architecture and Implementation

**Figure 3:**
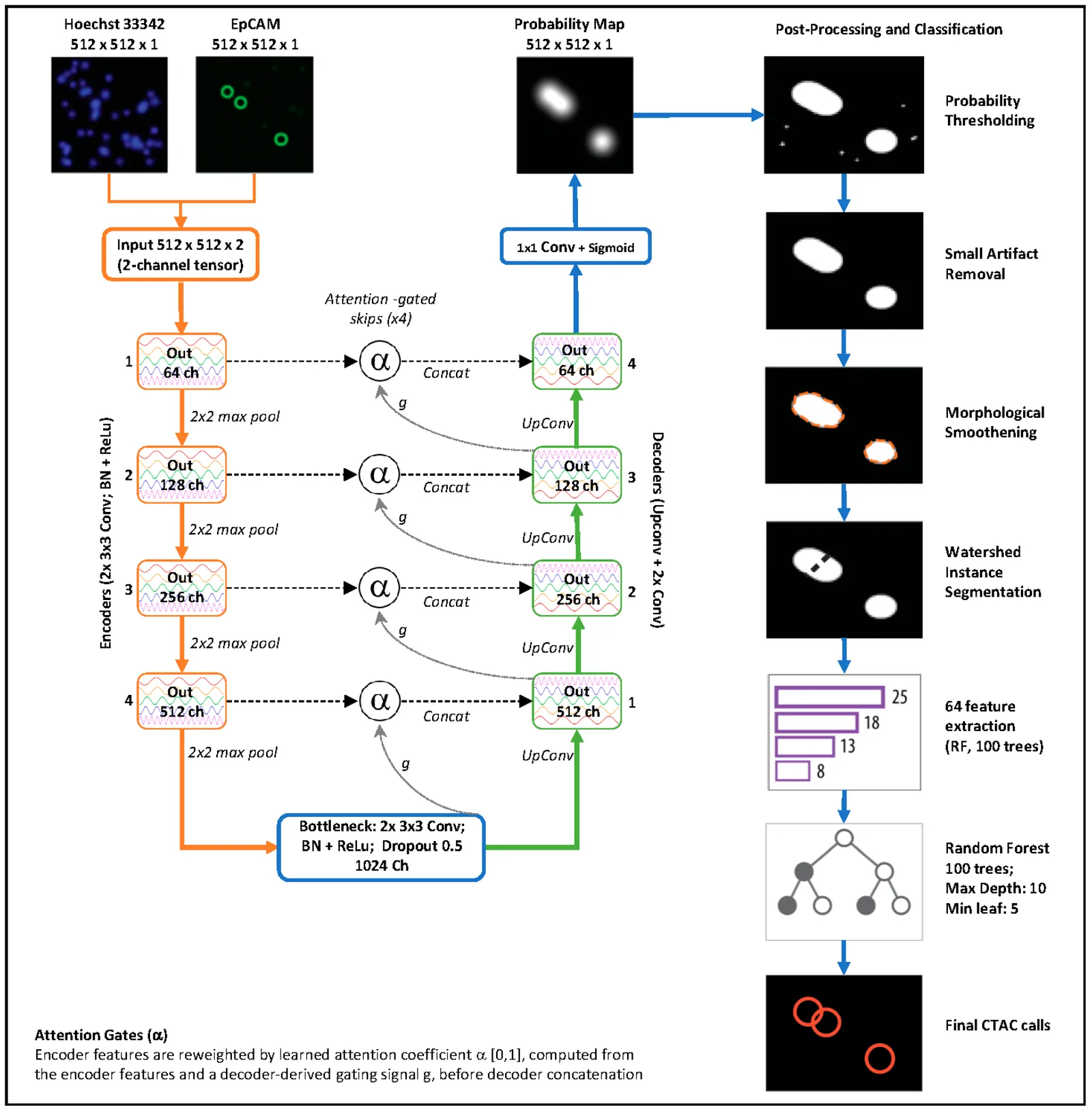
Architecture of the predefined integrated CTAC-detection framework. Dual-channel Hoechst/EpCAM input enters an encoder–decoder Attention U-Net with attention-gated skip connections and sigmoid probability-map output, followed by thresholding, artifact removal, morphological smoothing, watershed instance segmentation, 64-feature extraction, and Random Forest classification. The attention-gating principle follows established Attention U-Net architecture. The schematic panels are illustrative only and not to scale.

The segmentation module used an Attention U-Net architecture for CTAC detection from dual-channel EpCAM/Hoechst 33342 fluorescence images (Figure 3), followed by CTAC-specific post-processing, feature extraction, and Random Forest classification. The network processes 512 × 512-pixel inputs through an encoder–decoder framework with skip connections. The encoder uses 4 blocks of dual 3 × 3 convolutions (with rectified linear units, ReLU (Rectified Linear Unit), and batch normalization) followed by 2 × 2 max pooling, which expands the total channels from 64 to 1024. A bottleneck with two convolutions and dropout (0.5) provides regularization. The decoder mirrors this structure with 4 blocks of transposed convolutions, skip connections, and dual convolutions, reducing channels from 512 to 64. A final 1 × 1 convolution with sigmoid activation generates pixel-wise CTAC probability maps. In this model, the attention gates in the skip connections highlight relevant spatial features before decoder concatenation.

Each attention gate combines an encoder feature map x^l^ with a coarser gating signal g from the decoder using learned linear transformations, ReLU activation, and a sigmoid attention coefficient α^l^ in the interval [0, 1]. Here, the attention coefficient (αl) was computed as α^l^ = **σ**$\left(W_{x}^{T}x^{l}+W_{g}^{T}g+b_{g}\right)$**,** where ‘x^l^’ is the feature from the encoder at layer ‘l’, g is the gating signal from the decoder, $\left(W_{x}^{T}x^{l}+W_{g}^{T}g+b_{g}\right)$ is the linear combination for attention, and ‘σ’ is typically a sigmoid function that yields attention coefficients between 0 and 1. Next, the encoder features were reweighted via element-wise multiplication, as $x^{\hat{l}}$ = *x^l^* ⊙ *α^l^*. The gated feature is then passed through the skip connection for decoder concatenation, following the established Attention U-Net formulation [18,19]. In this manner, the attention gate emphasizes image regions that are more likely to contain CTAC signals, while simultaneously down-weighting regions with noise (e.g., intact PBNCs or cellular debris); i.e., the model focuses its segmentation effort on higher CTAC-probability regions.

### 2.8. Model Training

Model development data were partitioned into training (70%), validation (15%), and testing (15%) subsets at the independent biological-source level before image-level augmentation. All images, fields of view, cellular objects, and derivatives originating from the same patient/donor source were retained within a single partition; no biological source contributed data to more than one partition. A source-lineage audit confirmed zero patient/donor overlap among the training, validation, and test sets. The extreme class imbalance (i.e., 1 CTAC per ≥ 10^6^ blood cells) was addressed by applying a weighted loss approach, coupled with extensive data augmentation. Model training employed a hybrid objective, combining binary cross-entropy and Dice loss (α = 0.3), optimized using Adam (initial learning rate = 10^−4^; decay factor 0.9 every 5 epochs). Each batch comprised 16 image patches, with early stopping (patience = 15) guided by validation loss. Hyperparameters, including learning rate, weight decay, dropout rate, batch size, and loss-weighting were optimized through Bayesian optimization with 5-fold cross-validation. To improve convergence, we applied cosine annealing with warm restarts [22], starting with 10-epoch cycles and doubling thereafter. Our augmentation pipeline combined both class-aware and generic transformations. Synthetic CTAC images were generated by a conditional GAN (Generative Adversarial Network) [23,24,25] and used exclusively for minority-class augmentation within the training partition. Neither the data used to train the conditional GAN nor the GAN-generated images entered the model validation or test partitions. A subset of synthetic images underwent pathologist review for biological plausibility before use as training augmentation; this review constituted quality control of synthetic training material and not model validation. Generalization was further enhanced through standard augmentations such as rotations, flips, elastic deformations, contrast optimization, and noise modulation. For the final evaluation, we ensembled the best-performing models from 5-fold validation using majority voting to generate consensus segmentation masks.

### 2.9. Post-Processing and CTAC Classification

The U-Net output was refined through a structured post-processing pipeline that included probability thresholding, small artifact removal, morphological smoothing, and watershed-based instance segmentation to separate adjacent cells. Classification was performed in two integrated stages combining U-Net segmentation with a random forest model. In the first stage (Feature Extraction), a total of 64 features were computed for each segmented candidate: morphological (25, e.g., area, perimeter, solidity, eccentricity, axis lengths, and convexity), intensity-based (18, mean/median/max intensity, contrast, and entropy in EPCAM/Hoechst 33342 channels), texture-related (13, Haralick descriptors such as energy, correlation, and homogeneity), and spatial context (8: proximal intercellular distances and local density) [26,27]. In the second stage (Classification), the 64-dimensional feature vector feeds into a random forest classifier (100 trees, max depth = 10, min samples per leaf = 5) using the entropy criterion and class weights that are inversely proportional to class frequencies [28,29,30,31]. The Random Forest training set was independent of the U-Net training set. CTACs were identified based on defined criteria including cell diameter (>10 μm), EPCAM signal-to-background ratio (>2.5), nuclear-to-cytoplasmic ratio (>0.7), and characteristic epithelial morphology.

### 2.10. Analytical Validation

To ensure suitability for clinical use, we conducted comprehensive analytical validation of the qualitative CTAC detection model. The validation protocol assessed critical performance parameters, including the limit of detection, analytical specificity against commonly encountered non-malignant cells, precision (repeatability and reproducibility), sample stability, and potential interference from exogenous substances. These validation studies were aimed at establishing the analytical performance characteristics required for clinical implementation. Detailed protocols are provided in the Supplementary Materials.

### 2.11. Exploratory Study

**Figure 4:**
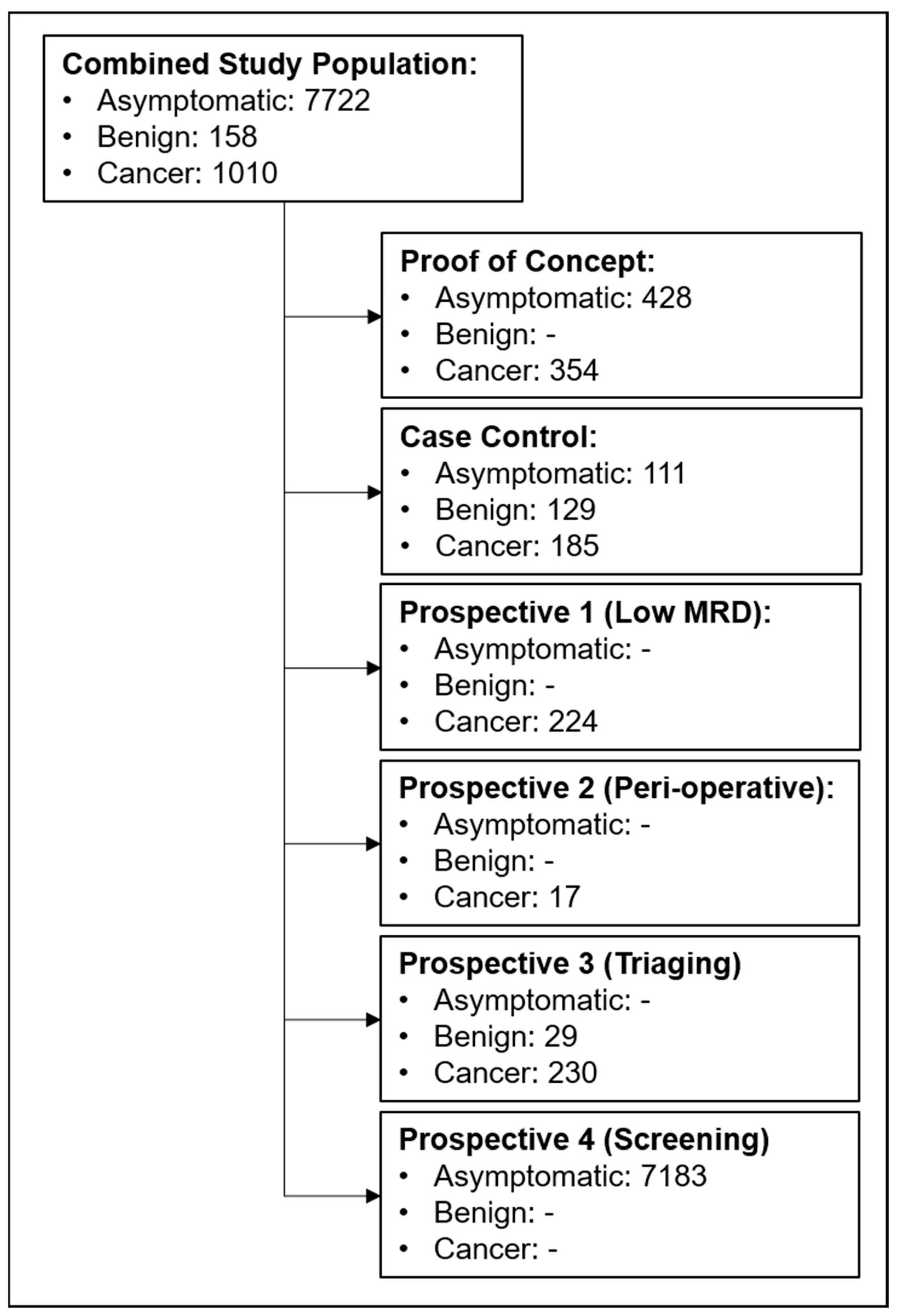
Clinical study cohorts. Diagrammatic representation of the overall study population as well as sub-cohorts.

The objective of the study was to demonstrate that CTACs are detected in advanced cancers and that CTACs are undetectable (or rarely detected) in asymptomatic individuals, i.e., the sensitivity and specificity of the approach. This study included peripheral blood specimens from asymptomatic individuals (n = 428) and patients (n = 354) with advanced, recurrent solid tumors. Demographics of the sub-cohorts are provided in Table 1. All blood specimens were processed as per the established workflow to determine the detection of CTACs. The Proof of Concept (PoC) study sub-cohorts were stratified into training, validation and test sets and evaluated as described in a previous section. Figure 4 depicts the various clinical study (sub-)cohorts.

### 2.12. Clinical Studies

The clinical performance of the integrated framework was evaluated in one case–control cohort and four distinct prospective cohorts addressing different intended use settings. These cohort evaluations were conducted using the same core laboratory, imaging, and analysis workflow.

#### 2.12.1. Case–Control Study

The study cohort (Table 1 and Table 2) included stage I/II therapy-naive cancer patients (n = 185), patients with benign conditions (n = 129), and asymptomatic volunteers with no history of cancer diagnosis (n = 111). With 185 cancer cases and 240 non-cancer controls, the study size allowed overall performance to be estimated with single-digit percentage precision. For a 90% estimated sensitivity, the 95% confidence interval (CI) was 85.7–94.3%, and for a 95% estimated specificity, the 95% CI was 92.2–97.8%, both indicating acceptable sensitivity and specificity estimation.

#### 2.12.2. Prospective Study 1 (Low Radiological Tumor Disease)

The study cohort (Table 1) included 224 patients with various solid tumors showing objective (complete or significant) response to anticancer treatments. The study determined the sensitivity of the model, i.e., the ability to detect tumors with lower tumor burden.

#### 2.12.3. Prospective Study 2 (Perioperative CTAC Dynamics)

This study evaluated peri-operative CTAC detectability in patients with solid tumors (Table 3) to examine whether CTAC detection changed temporally after surgical tumor resection. Each patient served as their own paired control. For this study, pre-surgery (−4 h) and post-surgery (+24 h) blood specimens were collected from recently diagnosed, systemic therapy-naive patients (n = 17) with resectable solid tumors and evaluated.

#### 2.12.4. Prospective Study 3 (Cancer Versus Benign Specificity)

The study cohort (Table 1) included 259 individuals suspected of various cancers based on clinical findings or symptoms. All patients underwent standard-of-care (SoC) tissue-based histopathological diagnosis. The objective of the study was to determine the ability of the model to support diagnostic triaging in suspected cancer cases.

#### 2.12.5. Prospective Study 4 (MCED in Cancer Screening Population)

The study cohort (Table 2) included 7183 asymptomatic individuals; 3218 males and 3965 females with a median age of 46 years (range: 20–94 years) who had availed the sponsor’s CTAC-based multi-cancer early detection (MCED) test. Leftover specimens were processed as per the described method. Individuals with CTAC positive findings (as per the approach described in this manuscript) were clinically followed up to determine cancer diagnosis. The objective of this study was to evaluate the suitability of the model for MCED in an unselected cancer screening population.

## 3. Results

### 3.1. Analytical Validation

Findings of the analytical validation studies are provided in the Supplementary Materials.

### 3.2. Exploratory Study

In this study, the model achieved an overall 90.68% sensitivity (95% CI: 87.16%, 93.50%) for CTAC detection in advanced cancers (Table 3). The sensitivity ranged from 66.67% (Head and Neck cancers) to 100% (Gastric, Uterine, Esophageal, Prostate, Kidney, Hepatobiliary). CTACs were undetectable in 99.53% (95% CI: 98.32%, 99.94%) asymptomatic individuals with no history of cancer diagnosis. The study findings established acceptable sensitivity and specificity of the approach.

### 3.3. Clinical Studies

#### 3.3.1. Case–Control Study

In the case–control study, the model demonstrated 88.65% sensitivity (95% CI: 83.17%, 92.83%) for detecting Stage I/II cancers (Table 3). The sensitivity ranged from 83.33% (Pancreatic cancer) to 100% (Prostate cancer). In this study also, lower CTAC-detection rate was also observed for Head and Neck cancer (84.21%). In this cohort, CTAC detection rate increased with grade, with 75% sensitivity in Low Grade (well-differentiated, n = 20), 87.93% sensitivity in Moderate Grade (moderately differentiated, n = 51), and 97.5% sensitivity in High Grade (poorly or undifferentiated, n = 40) tumors. There were no significant differences in CTAC detection rates based on gender (87.5% in males vs. 89.9% in females). Among the individuals with benign conditions, the model had 78.95% specificity (95% CI: 71.03%, 85.53%), with CTACs being undetectable in 105 of 129 cases. CTACs were detected in 15/46 (32.61%) cases of benign prostate conditions. The biological and clinical significance of these positive findings could not be determined because definitive longitudinal follow-up was unavailable. CTACs were undetectable in all specimens from asymptomatic individuals, yielding 100% specificity (95% CI: 96.73%, 100%). The findings of the case–control study suggest robust clinical performance characteristics that are required to support the intended clinical utility of the model.

#### 3.3.2. Prospective Study 1 (Low Tumor Burden)

The first prospective cohort included diagnosed and pretreated solid tumor cases (n = 224) with complete or significant treatment response (post SoC). The objective of this study was to determine the utility of the model for non-invasive post-treatment monitoring of disease status in patients with radiologically low or undetectable tumor burden. In this cohort of patients, the model achieved 91.96% CTAC-detection sensitivity (95% CI: 87.60%, 95.17%). CTAC detection sensitivity ranged from 87.5% (Colorectal) to 100% (Pancreaticobiliary, Gastroesophageal, Uterine, Prostate). The detection sensitivity was comparable in females (93.10%, n = 145) and males (91.14%, n = 79).

#### 3.3.3. Prospective Study 2 (Perioperative CTAC Dynamics)

In the perioperative study, CTACs were detected in all (n = 17) pre-surgery blood specimens. In the post-surgery specimens, CTACs were detectable in 5 cases (29.41%) and undetectable in 12 cases (70.59%). These paired observations demonstrate a temporal association between surgery and reduced postoperative CTAC detectability.

#### 3.3.4. Prospective Study 3 (Cancer Versus Benign Specificity)

**Figure 5:**
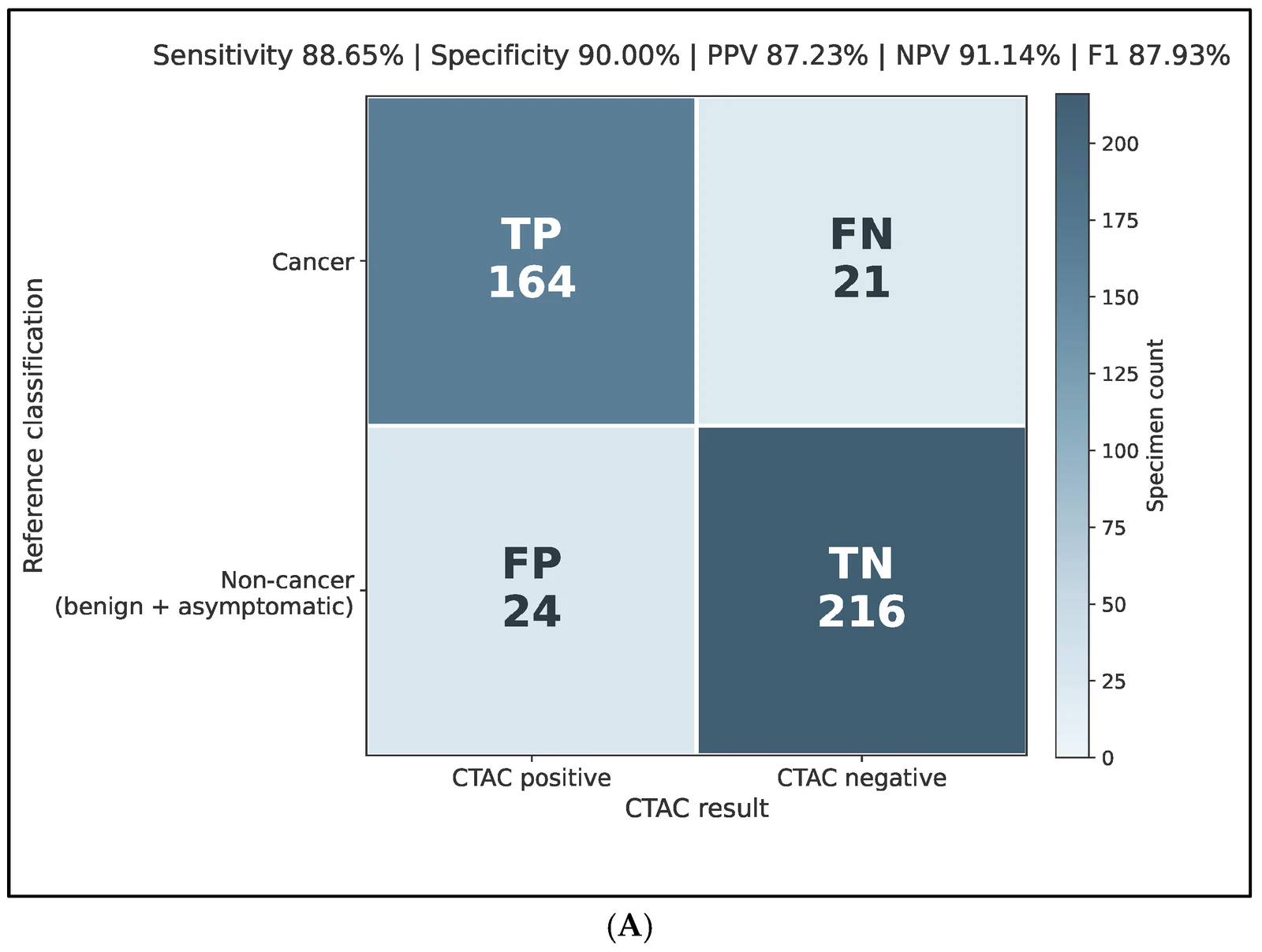

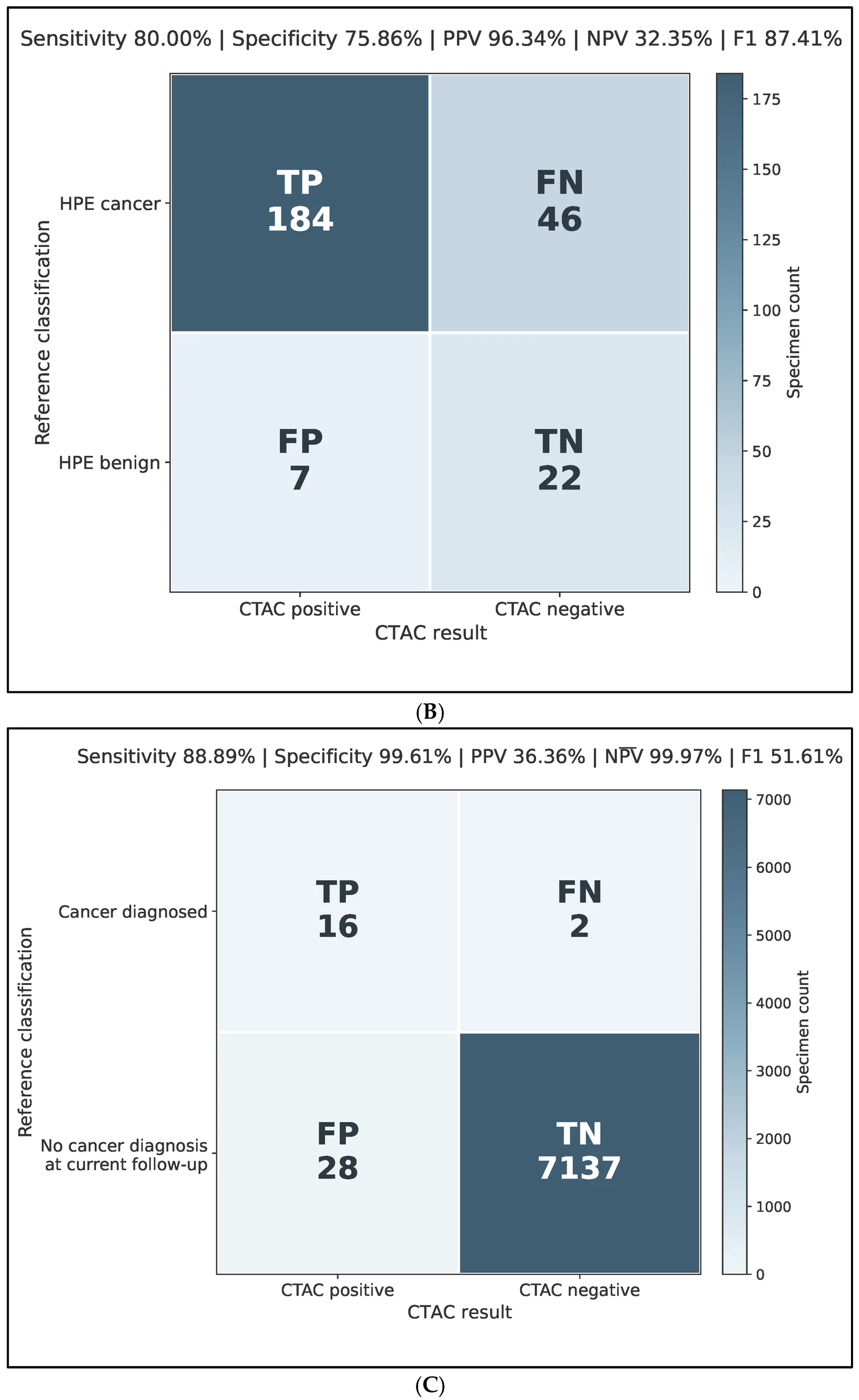
Confusion matrices summarizing specimen-level CTAC classification performance across three study cohorts. (**A**) Shows the case–control cohort, in which the non-cancer reference group combines benign conditions and asymptomatic controls. Note: Non-cancer controls combine benign (n = 129) and asymptomatic (n = 111) participants. (**B**) Shows the prospective suspected-cancer cohort, using histopathology as the reference standard. (**C**) Shows the MCED screening cohort, in which the displayed counts and performance values are provisional because unresolved CTAC-positive cases were provisionally treated as false positives at the time of analysis. In each matrix, rows represent the reference classification and columns represent the CTAC result.

This prospective cohort included patients (n = 259) with no prior diagnosis of cancer, who presented with clinical symptoms, clinical laboratory findings, or radiological findings suspected of cancer necessitating the standard of care (SoC) diagnostic pathway based on histopathological examination (HPE) of tissue sample. In this cohort, 191 patients were CTAC-positive, and 68 patients were CTAC-negative. Of the 259 suspected cases, histopathological diagnosis confirmed cancer in 230 cases and indicated benign conditions in 29 cases. When the CTAC detection status was correlated with histopathological diagnosis, the model demonstrated 96.34% PPV (95% CI: 93.22%, 98.05%) and 32.35% NPV (95% CI: 25.58%, 39.95%), based on a sensitivity of 80.00% (95% CI: 74.24%, 84.97%) and a specificity of 75.86% (95% CI: 56.46%, 89.70%) (Table 3). Given the enriched cancer prevalence in this cohort (88.8%), PPV and NPV were recalculated at an assumed cancer prevalence of 25% to reflect a more general clinical setting. Under this assumption, PPV and NPV were 52.49% (95% CI: 36.61%, 67.88) and 91.92% (95% CI: 89.11%, 94.06%), respectively. At the specimen level, the histopathology-referenced confusion matrix comprised 184 true-positive, 46 false-negative, 7 false-positive, and 22 true-negative CTAC results (Figure 5). This corresponds to a sensitivity of 80.00%, specificity of 75.86%, PPV of 96.34%, NPV of 32.35%, and F1 score of 87.41%.

#### 3.3.5. Prospective Study 4 (MCED in Cancer Screening Population)

Among the 7183 asymptomatic individuals, 7139 (99.39%) were CTAC negative and 44 (0.61%) were CTAC positive. Among the 7139 CTAC-negative cases, 2 (0.03%) were diagnosed with Stage 0/I cancers. Among the 44 CTAC-positive cases, 16 (36.4%) were diagnosed with Stage I/II cancers, including prostate (n = 6), breast (n = 3), colon (n = 2), cervix (n = 2), stomach (n = 2), or esophagus (n = 1). In 10 CTAC-positive cases, there was no radiologically detectable evidence of disease.

Follow-up was incomplete for 18 cases that remained unresolved at the time of submission. These unresolved cases were not classified as definitive false positives. Under a conservative lower-bound analysis in which all 18 unresolved CTAC-positive participants are assumed not to have cancer, sensitivity is 88.89%, specificity is 99.61%, PPV is 36.36%, and NPV is 99.97%. If all 18 unresolved participants were subsequently confirmed to have cancer, the corresponding boundary estimates would be sensitivity 94.44%, specificity 99.86%, PPV 77.27%, and NPV 99.97%. These estimates are provisional.

Confusion matrices (Figure 5) were generated to summarize specimen-level classification performance in the case–control, prospective suspected-cancer, and MCED screening cohorts. In the case–control cohort, the combined non-cancer reference group comprised individuals with benign conditions and asymptomatic controls. In the suspected-cancer cohort, histopathological diagnosis served as the reference standard. For the MCED screening cohort, the matrix was considered provisional because 18 CTAC-positive individuals remained unresolved at the time of analysis and were provisionally classified as false positives. The matrices provide a direct visualization of true-positive, false-negative, false-positive, and true-negative classifications and complement the corresponding sensitivity, specificity, PPV, NPV, and F1 estimates.

## 4. Discussion

This study evaluates the performance of a predefined integrated CTAC-detection framework combining Attention U-Net segmentation, CTAC-specific post-processing, cytological feature extraction, and Random Forest classification. The case–control and prospective cohort findings support further evaluation of the framework across cancer detection and monitoring settings. Because the attention mechanism follows established Attention U-Net principles and no cross-model benchmarking was undertaken, the results are interpreted as the performance of the integrated framework rather than evidence of architectural superiority.

Conventional CTC assays have been constrained by low sensitivity, low specificity, lack of harmonized standards for interpretation, and inadequately established clinical utility value or actionability, all of which may have contributed to a gradual decline in enthusiasm for CTCs as robust clinical biomarkers [3,4,32,33]. The historical limitations that may have undermined the reliability of CTC-based technologies arise from methodological and analytical constraints and not from the biological characteristics of CTCs themselves [3,34]. Our framework leveraged pixel-level segmentation (rather than bounding box-based detection) to achieve precise characterization of cellular and nuclear morphology based on biomarker localization (fluorescence signal) patterns. The framework incorporated attention-gated skip connections to provide learned spatial weighting before decoder concatenation, together with CTAC-specific post-processing and feature-based classification. The observed performance is attributable to the integrated pipeline as evaluated; the present study was not designed to quantify the marginal contribution of attention gates, augmentation, post-processing, or the Random Forest individually. The performance across diverse cancer types suggests that the model captured generalizable morphological and fluorescence (biomarker positivity and localization) features of CTACs and was not prone to confounding effects of tumor cancer-specific variabilities. Our model uses predefined size exclusion and nuclear-to-cytoplasmic (N:C) ratio parameters in conjunction with biomarker (EPCAM) positivity for CTAC detection. While EPCAM status provides molecular specificity to confirm epithelial tumor (but not the organ of origin), the biophysical and morphological filters provide orthogonal verification to improve detection accuracy [35,36]. Together, they reduce false positives from large or atypical leukocytes and yield a more robust and reliable identification of CTACs across heterogeneous tumor states. This model addresses several key analytical limitations that have undermined other approaches. The findings demonstrate that meaningful technological refinement can yield objective gains in accuracy and reproducibility, which reaffirm the credibility of CTACs/CTCs as a reliable biomarker in oncology.

The framework was evaluated in case–control and prospective cohorts spanning different clinical contexts. Consistency across these cohorts supports the relevance of the integrated workflow within the study setting; however, all evaluations used the same core laboratory/imaging process and do not establish transportability across independent laboratories, scanners, or populations [37].

The peri-operative cohort showed a marked reduction in CTAC detectability 24 h after surgical resection compared with the pre-surgery sample in the same patients. This temporal association is biologically compatible with a relationship between tumor presence and CTAC detection, but the small paired cohort was not intended to establish causality [38,39].

The attention-gating mechanism used here follows established Attention U-Net principles [18,19]. The downstream classification stage is feature-definitive: each segmented candidate is represented by 64 predefined morphological, intensity, texture, and spatial-context variables before Random Forest classification. This structure provides a transparent mapping between the classifier input and biologically recognizable cytological attributes. The archived revision materials do not contain the trained Random Forest object with the held-out feature matrix or executable U-Net activation outputs required to reconstruct reliable feature-importance, attention-map, or saliency analyses for the originally locked model.

The U-Net’s encoder–decoder structure is well-matched to the detection problem. The encoder progressively compresses spatial information through convolutional and pooling layers, building increasingly abstract feature representations at each level—from fine details such as membrane-localized EPCAM fluorescence and nuclear texture at shallow layers to broader cellular geometry and size relationships at deeper layers. The decoder reconstructs this back to full resolution for precise cell boundary delineation. This multi-scale hierarchy maps naturally to the definition of CTACs: their identification rests on features at different spatial scales, from EPCAM signal at the membrane, through nuclear morphology, to the N:C ratio. Skip connections preserve multi-scale encoder information for the decoder, while attention gates provide learned spatial weighting of these transferred features. These architectural properties are established features of Attention U-Net and are described here to explain model structure, not to claim component-specific performance gains in the absence of ablation analysis.

The evaluated cohorts correspond to several potential clinical contexts, including early-stage cancer detection, diagnostic triaging, post-treatment assessment, and screening. In the suspected-cancer cohort, the high PPV occurred in a population with very high cancer prevalence and should therefore be interpreted as cohort-specific rather than as a general rule-in performance estimate. In the asymptomatic cohort, the high NPV is likewise prevalence-dependent and the positive arm remained incompletely adjudicated [40]. CTAC-positive findings should remain subject to conventional diagnostic confirmation, and CTAC-negative individuals should continue recommended standard-of-care screening [41].

In addition to the perceived benefits, we acknowledge the following possible limitations. The reliance on EPCAM as the sole epithelial marker may overlook CTACs with low or no epithelial features (e.g., mesenchymal phenotypes), potentially impacting detection (sensitivity) [42,43]. While this has been a limitation of EPCAM-based CTAC immuno-enrichment methods, EPCAM immunofluorescence staining has higher sensitivity for CTAC detection. We intend to evaluate additional biomarkers to improve CTAC detection and reduce residual false-negative risks. The present study focuses on epithelial malignancies, and its utility or performance in non-epithelial cancers is not yet established. The case–control clinical study design may overestimate real-world performance compared to a prospective screening context [44]. However, this is partially mitigated by consistent results from the inclusion of unselected populations. The PPV observed in the cancer screening cohort (n = 7183) reflects the rarity of early-stage cancers in asymptomatic populations and highlights the importance of follow-up confirmation using imaging or other diagnostic modalities [41]. The incomplete longitudinal outcome data for the screening cohort participants limits definitive assessment of long-term clinical benefit and interval cancer incidence [45]. The study was designed around the final qualitative CTAC call. The uploaded archival record available for this revision does not contain paired pixel-level ground-truth and locked-model prediction masks required to calculate Dice, IoU, pixel precision, pixel recall, object-level CTAC recall, or false-positive objects per field without rerunning or reconstructing the original evaluation. Similarly, the model artifacts required for retrospective Random Forest feature-importance and U-Net saliency/attention analyses are not available in the revision record. In addition, the present prospective cohorts were processed within the same core workflow.

Tumor burden was not operationalized using rigid quantitative thresholds, and classification into low- or high-burden categories therefore relied on clinical (or radiological) interpretation. The model is currently intended for qualitative CTAC detection, and not to quantitatively correlate CTACs with tumor volume or disease kinetics. The model is currently not intended to provide information on the organ or tissue of origin (cancer type and subtype) in any application, including MCED. The primary objective of MCED is to identify biologically significant malignancy at an early stage to improve clinical outcomes. Hence, a more appropriate metric for evaluating MCED would be sensitivity for Stage I/II disease. Tissue localization is clinically informative but contingent on successful detection and depends on imaging and histopathological confirmation. Methods that rely predominantly on tumor-derived molecular shedding, including methylation-based assays, may have lower performance in early-stage and low-shedding cancers, where analyte abundance is constrained [46,47]. In contrast, detection of systemic signals of malignancy could potentially enable timely diagnostic escalation even when anatomical attribution is indeterminate. This hierarchy preference of detection over localization aligns with real-world expectations and oncology workflows.

## 5. Conclusions

A predefined integrated pipeline combining Attention U-Net segmentation, CTAC-specific post-processing, cytological feature extraction, and Random Forest classification demonstrated consistent CTAC detection across the evaluated case–control and prospective cohorts. The attention mechanism follows established Attention U-Net principles, and the present study does not establish superiority over alternative architectures or the independent contribution of individual components. The findings support the potential clinical utility of CTAC-based image analysis for cancer detection and monitoring settings in synchronicity with the Standard of Care.

## Figures and Tables

**Table 1:** Study-wise demographics of patients with cancer and non-malignant (benign) conditions.

Study		Proof of Concept	Case–Control		Low Tumor Burden	Peri- Operative	Suspected Cases (Final Diagnosis)	
Type		Cancer	Cancer	Benign	Cancer	Cancer	Cancer	Benign
n =		354	185	129	224	17	230	29
Gender								
	Male	115	88	83	79	11	80	11
	Female	239	97	46	145	6	150	18
Age (years)								
	Median	57	55	60	64	52	53	50
	Range	24–88	20–83	17–88	21–88	26–76	19–92	17–77
Organ							-	
	Breast	98	51	8	54	1	82	8
	Colorectal	83	22	-	56	5	12	-
	Lung	26	1	4	23	-	23	4
	Ovary	34	7	16	21	-	24	6
	Pancreas	26	6	12	18	-	11	-
	Prostate	9	9	46	5	-	14	6
	Head and Neck	6	57	2	2	7	33	2
	Esophagus	9	1	-	5	-	1	-
	Uterine	12	7	-	6	1	3	-
	Gastric	12	5	-	7	-	1	-
	Unknown	10	1	31 *	3	-	3	-
	Kidney	5	3	1	4	-	4	-
	Hepatobiliary	12	-	3	7	-	4	2
	Bladder	2	-	-	3	-	3	-
	Cervix	1	4	1	-	2	10	1
	Thyroid	4	5	5	3	-	1	-
	Thymus	2	-	-	2	-	1	-
	Anal	2	-	-	2	-	-	-
	Penis	1	3	-	1	-	-	-
	Small bowel	-	1	-	2	-	-	-
	Vulva	-	2	-	-	1	-	-

* includes conditions such as stroke, diabetes, hypertension, pyrexia or infection.

**Table 2:** Study-wise demographics of asymptomatic individuals.

Study		Proof of Concept	Case–Control	Cancer Screening
n =		428	111	7183
Gender				
	Male	214	63	3218
	Female	214	48	3965
Age (years)				
	Median	46	44	46
	Range	20–83	20–94	20–94

**Table 3:** CTAC detection in cancers. Consolidated CTAC detection rates observed in various cancer cases across all clinical study cohorts. Where the number of any cancer type in any study is <5, the findings are grouped under ‘Others’.

Cancer Type	CTAC Positive Rates (n/N, %) in Cancers in Various Study Cohorts			
	Proof of Concept	Case–Control	Low-Tumor Burden	Prospective
Breast	88/98 (89.80%)	44/51 (86.27%)	50/54 (92.59%)	75/82 (91.46%)
Colorectal	72/83 (86.75%)	21/22 (95.45%)	49/56 (87.50%)	9/12 (75.00%)
Ovary	32/34 (94.12%)	6/7 (85.71%)	19/21 (90.48%)	17/24 (70.83%)
Lung	24/26 (92.31%)	-	21/23 (91.30%)	17/23 (73.91%)
Pancreas	24/26 (92.31%)	5/6 (83.33%)	18/18 (100%)	9/11 (81.82%)
Gastric	12/12 (100%)	-	7/7 (100%)	-
Uterine	12/12 (100%)	6/7 (85.71%)	6/6 (100%)	-
Unknown primary	8/10 (80.00%)	-	-	-
Esophagus	9/9 (100%)	-	5/5 (100%)	-
Prostate	9/9 (100%)	9/9 (100%)	5/5 (100%)	13/14 (92.86%)
Hepatobiliary	12/12 (100%)	-	5/5 (100%)	-
Head and neck	4/6 (66.67%)	48/57 (84.21%)	-	21/33 (63.64%)
Kidney	5/5 (100%)	-	-	-
Cervix	-	-	-	8/10 (80.00%)
Others	10/12 (83.33%)	25/26 (96.15%)	21/24 (87.5%)	14/21 (66.67%)
Total	321/354 (90.68%)	164/185 (88.65%)	206/224 (91.96%)	184/230 (80.00%)

CTAC—Circulating Tumor Associated Cell; n—Number of subjects tested positive; N—Total number of subjects.

## Data Availability

More comprehensive descriptions of the U-Net structure and the details on hyperparameter training can be made available on request under appropriate non-disclosure agreements.

## Supplementary Materials

The following supporting information can be downloaded at: https://www.mdpi.com/article/10.3390/cancers18162691/s1, Summary of Analytical Validation; Table S1: Reagents, Consumables and Standard Cell Lines.

## Abbreviations

AI	Artificial Intelligence
CI	Confidence Interval
CDX2	Caudal Type Homeobox 2, colon marker
CNN	Convolutional Neural Network
CTAC	Circulating Tumor Associated Cell
DCIS	Ductal Carcinoma In Situ
EpCAM	Epithelial Cell Adhesion Molecule
FOV	Field of View
GAN	Generative Adversarial Network
GATA3	Transcription factor, breast marker
H&E	Hematoxylin and Eosin (staining)
ICC	Immunocytochemistry
MCED	Multi-Cancer Early Detection
NC	Negative Control
NKX3.1	Homeobox gene, prostate marker
NPV	Negative Predictive Value
PAX8	Paired Box Gene 8, ovary marker
PBNC	Peripheral Blood Nucleated Cell
PBS	Phosphate-Buffered Saline
PC	Positive Control
PPV	Positive Predictive Value
RBC	red blood cell
ReLU	Rectified Linear Unit
SOX9	SRY-Box Transcription Factor 9, pancreas marker
STR	Short Tandem Repeat
TTF1	Thyroid Transcription Factor 1, lung marker
