# Supplementary material for: Attention U-Net-Based Segmentation and Hybrid Classification for Detection of Circulating Tumor-Associated Cells
Source: Cancers (Basel). 2026 Aug 20;18(16):2691. doi: 10.3390/cancers18162691 (PMC13511424; doi:10.3390/cancers18162691)
Supplement: Supplementary file 1 [file cancers-18-02691-s001.zip › cancers-4474411-supplementary.pdf]

**Summary of Analytical Validation**

The analytical performance characteristics of the process was evaluated in structured studies.

**Accuracy:** Accuracy was established on contrived specimens generated using MCF7, breast cancer cell line that shows consistent high EpCAM positivity. Peripheral blood collected from healthy donors (HD; n = 15; 9 male, 6 female; median age 38 years, range 24-56 years) was used for this study. Fifteen 1 mL aliquots 1 each from all 15 individuals) were unspiked, fifteen 1 mL aliquots (1 each from all 15 individuals) were spiked with 100 MCF7 cells, and five 1 mL aliquots (1 each from 5 individuals) were spiked with 50 MCF7 cells. All samples were processed as per the test workflow for detection of EpCAM+ cells. The prespecified acceptance criteria were >90% positive percent agreement (PPA) and >95% negative percent agreement (NPA). EpCAM+ cells were detected in all spiked specimens (n = 20), while no marker-positive cells were detected in unspiked specimens (n = 15). Based on this, the test had a PPA of 100% (95% CI: 83.16%, 100.00%), NPA of 100% (95% CI: 78.20%, 100.00%), and overall agreement of 100% (95% CI: 90.00%, 100.00%).

**Precision:** Precision was established using contrived specimens. generated using MCF7 (breast cancer) cell line that shows consistent high EpCAM positivity. HD peripheral blood pool was used for this study. HD blood samples (5 mL) were spiked with MCF7 cells to generate positive samples, while unspiked HD blood aliquots (5 mL) were used as negative samples. All samples were processed as per the test workflow for detection of EpCAM+ cells. A total of 77 positive samples and 77 negative samples were processed by 2 operators, over >20 days on a single instrument. Precision was established from the PPA and NPA in positive and negative samples respectively. The prespecified acceptance criteria were >95% PPA and >99% NPA. EpCAM+ cells were detected in all 77 positive samples yielding a PPA of 100% (95% CI: 95.32%, 100%). EpCAM+ cells were undetectable in all 77 negative samples yielding a NPA of 100% (95% CI: 95.32%, 100%).

**Specificity:** The Specificity of the test was established using contrived specimens. generated using SJCRH30, U87MG, and A375 cells that show consistent negative status for EpCAM. HD peripheral blood pool was used for this study. HD blood samples (5 mL) were spiked with 5, 10, 50, 100, and 200 of each type of cells (multiple replicates). All samples were processed as per the test workflow for detection of EpCAM+ cells. Specificity was established from the NPA. The prespecified acceptance

criteria was >99% NPA. EpCAM+ cells were undetectable in all samples yielding 100% NPA.

**Limit of Blank (LoB):** The Limit of blank of the test was established using HD blood samples from 10 volunteers (5 male, 5 female; age range 39-56 years) with no prior diagnosis of cancer and no current suspicion of cancer. 5 mL blood samples were collected and processed as per the test workflow for detection of EpCAM+ cells. The Limit of Blank for qualitative determination was established from the NPA in the known negative samples. The prespecified acceptance criteria was >99% NPA. EpCAM+ cells were undetectable in all negative samples yielding NPA of 100% (95% CI: 78.20%, 100.00%).

**Limit of Detection (LoD):** The Limit of detection of the test was established using contrived specimens. generated using MCF7 (Breast Cancer), SW620 (Colon Cancer), PL45 (Pancreatic Cancer), OVCAR3 (Ovarian Cancer), PC3 (Prostate Cancer) cell lines that show consistent high EpCAM positivity. HD peripheral blood pool was used for this study. HD blood samples (5 mL) were spiked with each type of cell lines at 1, 2, 5, 10, 50 and 100 cells with multiple replicates per spike level. All samples were processed as per the test workflow for detection of EpCAM+ cells. The LoD was determined as the lowest number of spiked cells that could be detected in at least 50% of the replicates. Based on these criteria, the LoD was determined to be 2 cells / mL.

The complete analytical validation datasets may be obtained from the authors upon request.

**Supplementary Table S1. Reagents, Consumables and Standard Cell Lines**

| Name | Manufacturer | Catalog Number | City | Country |
|---|---|---|---|---|
| BD Pharm Lyse™ Lysing Buffer | BD Biosciences | 555899 | San Jose | USA |
| Recombinant human Anti-CD326 (REA764)-PE-Vio-615 | Miltenyi Biotec | 130-111-006 | Bergish Gladbach | Germany |
| Hoechst 33342 (nuclear stain) | Thermo Fisher | 62249 | Waltham, Massachusetts | USA |
| ProLong Gold Mounting Medium | Invitrogen | P36930 | Carlsbad, California | USA |
| Superfrost Plus Microscopic slide | Thermo Fisher | 22037246 | Waltham, Massachusetts | USA |
| SKBR3 (Breast Cancer Cell Line) | ATCC | HTB-30 | Manassas, Virginia | USA |
| MCF7 (Breast Cancer Cell line) | ATCC | HTB-22™ | Manassas, Virginia | USA |
| SW620 (Colon Cancer Cell Line) | ATCC | CCL-227™ | Manassas, Virginia | USA |
| A549 (Lung Cancer Cell Line) | ATCC | CCL-185™ | Manassas, Virginia | USA |
| PL45 (Pancreatic Cancer Cell line) | ATCC | CRL-2558™ | Manassas, Virginia | USA |
| OVCAR3 (Ovarian Cancer Cell line) | ATCC | HTB-161™ | Manassas, Virginia | USA |
| PC3 (Prostate Cancer Cell line) | ATCC | CRL-1435™ | Manassas, Virginia | USA |
| SJCRH30 (Sarcoma Cell line) | ATCC | CRL-2061™ | Manassas, Virginia | USA |
| U87MG (Glioblastoma Cell line) | ATCC | HTB-14™ | Manassas, Virginia | USA |
| A375 (Melanoma Cell line) | ATCC | CRL-1619™ | Manassas, Virginia | USA |
